# Typhoid Fever Presenting With Cerebral Venous Thrombosis: A Report of a Rare Case

**DOI:** 10.7759/cureus.82534

**Published:** 2025-04-18

**Authors:** Amine Oulalite, Hamza Mimouni, Meriem Jartit, Housam Bkiyar, Brahim Housni

**Affiliations:** 1 Department of Anesthesia and Reanimation, Mohammed VI University Hospital, Faculty of Medicine and Pharmacy, Mohammed First University, Oujda, MAR; 2 Department of Neurology, Regional Hospital Center Al Farabi, Oujda, MAR

**Keywords:** cerebral venous thrombosis, fever, salmonella, thrombosis, typhoid fever

## Abstract

Typhoid fever, caused by *Salmonella typhi*, is a significant infectious disease in endemic regions. Although it primarily affects the gastrointestinal system, systemic complications, including vascular thrombosis, can occur. We present the case of a 47-year-old previously healthy woman who developed diarrhea, vomiting, and headache in the context of fever, complicated by altered consciousness. Contrast-enhanced computed tomography revealed sagittal sinus thrombosis. Blood cultures were negative for *Salmonella*, but Widal and Felix serologic testing was positive. The patient was successfully treated with antibiotics and anticoagulants. *S. typhi* can, in rare cases, lead to cerebral thrombotic events by inducing a hypercoagulable state through inflammation and endothelial dysfunction. This case highlights the rare association of *S. typhi *infection with cerebral venous thrombosis, emphasizing the importance of considering this etiology in similar clinical presentations.

## Introduction

Typhoid fever is a potentially severe systemic infection caused by *Salmonella typhi *(*Salmonella enterica *serotype Typhi), primarily spread through the fecal-oral route. It is most prevalent in developing countries where sanitation and hygiene conditions are inadequate. S. typhi and *Salmonella paratyphi* A, B, and C are the main pathogens responsible [1]. The worldwide incidence of typhoid fever is estimated at approximately 16 million cases annually, with a mortality rate of around 200,000 deaths each year [2]. Cerebral venous thrombosis (CVT) is a rare cause of stroke with multiple etiologies. Several general infectious conditions, including typhoid fever, have been associated with the occurrence of CVT [3,4]. We present the case of a 47-year-old female patient with no significant medical history who developed CVT, possibly due to a hypercoagulable state induced by *S. typhi* infection. The rare occurrence of this complication highlights the need to consider infectious diseases, such as *S. typhi*, in the evaluation of CVT, particularly after eliminating more common causes.

## Case presentation

A 47-year-old female patient, married and a mother of two children, was independently performing daily activities prior to her hospitalization in April 2022. She presented with a headache, vomiting, and watery diarrhea that began three days before admission, followed by the development of fever and altered consciousness. At the time of admission, her Glasgow Coma Scale score was 11 out of 15, with no signs of focal neurological deficits. Physical examination revealed a soft, non-distended abdomen. Laboratory tests revealed an inflammatory syndrome, with a C-reactive protein (CRP) level of 89 mg/L (normal range: <10 mg/L) and leukocytosis of 15,800/mm³ (normal range: 4,000-11,000/mm³). Blood cultures were negative, while serodiagnosis using the Widal and Félix tests showed elevated Anti BO (Proteus OX-2 antigen) antibodies (>100, normal range: <1:40) and negative Anti BH (Proteus OX-19 antigen) antibodies (normal range: <1:40) (Table 1). A lumbar puncture, including cytological and biochemical analysis, was normal.

**Table 1:** Laboratory investigations

Parameters	Patient Values	Reference Range
White Blood Cell Count (WBC)	15,800/mm³	4,000-11,000/mm³
C-Reactive Protein (CRP)	89 mg/L	<10 mg/L
Anti-BO Antibodies	>100	<1:40
Anti-BH Antibodies	Negative	<1:40
Blood Cultures	Negative	-
Cerebrospinal fluid (CSF) Analysis	Normal	-

**Figure 1:**
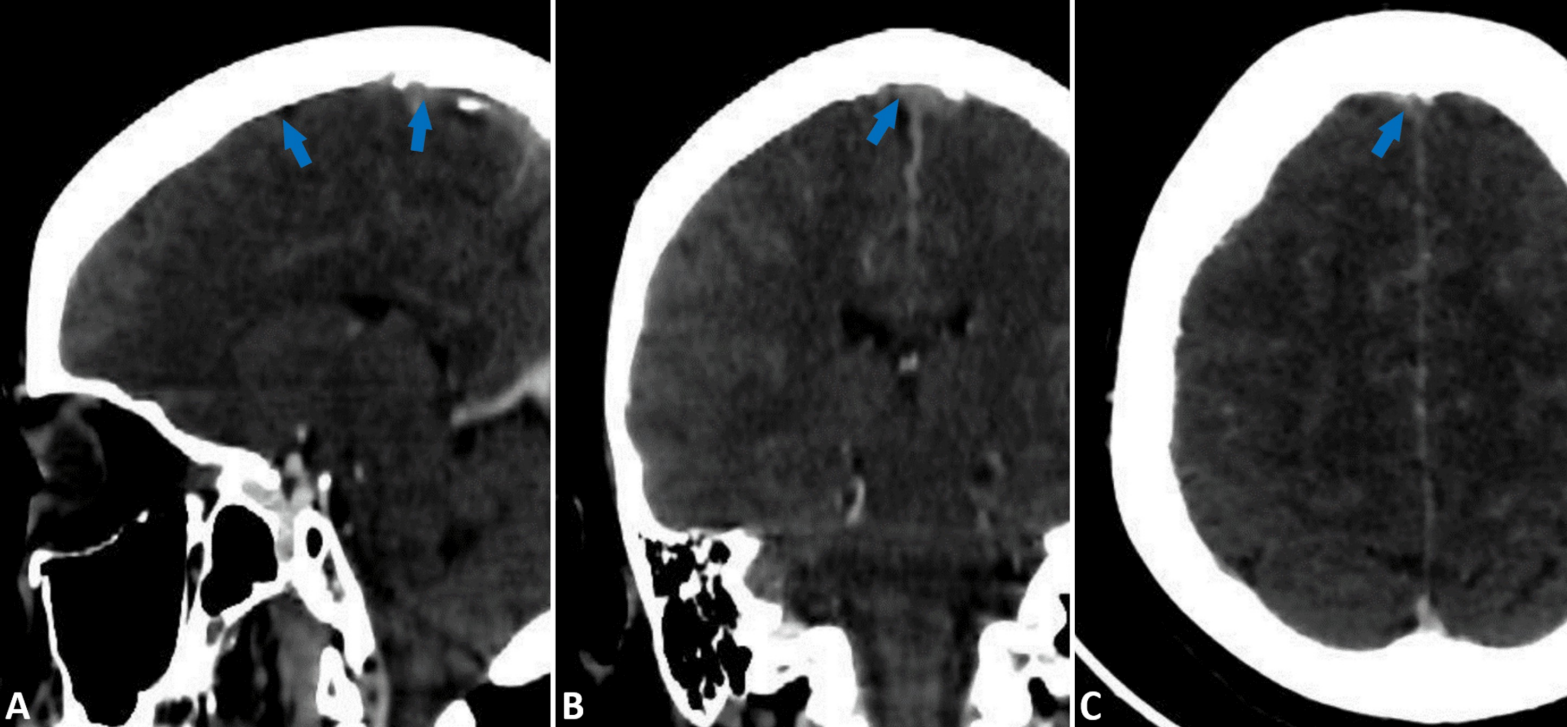
Contrast-enhanced cerebral CT scan in sagittal (a), coronal (b), and axial (c) views demonstrates the absence of opacification in the superior sagittal sinus, indicative of cerebral venous thrombosis (Blue arrows).

Initially, a non-contrast cerebral CT scan was performed, followed by contrast-enhanced CT imaging, which revealed sagittal cerebral thrombosis (Figure 1).

The patient was treated with ceftriaxone (70 mg/kg/day) and dexamethasone (0.4 mg/kg/day) for one week, followed by a gradual reduction in steroid dosage. The patient showed favorable progress, with complete resolution of neurological symptoms, and was discharged after a 13-day hospitalization. Curative anticoagulation therapy was continued for six months.

## Discussion

Typhoid fever is a systemic infection originating from the lymphatic system, with progressive spread through the bloodstream. Bacterial lysis within the mesenteric lymph nodes releases endotoxins, which contribute to systemic complications. Although vascular complications, particularly thromboembolic events like cerebral thrombosis, are extremely rare in typhoid fever, they have been reported [5].

The clinical presentation of typhoid fever varies widely, with onset ranging from acute to subacute or chronic. Neurological symptoms can range from mild headaches to deep coma, making diagnosis challenging. Brain imaging is the primary tool for confirming neurological involvement [6].

Typhoid fever has been linked to a variety of neurological complications [7]. Among its infectious manifestations, *Salmonella* meningoencephalitis is associated with a high risk of acute neurological conditions, including subdural abscesses, ventriculitis, encephalitis, hydrocephalus, venous thrombosis, and cerebral infarctions [8]. Non-infectious complications include neuropsychiatric disorders, cerebellar ataxia, encephalopathy, and encephalomyelitis [9].

With the widespread use of antibiotics, infectious causes of CVT have become uncommon, now accounting for less than 10% of cases [10]. Anticoagulation therapy remains the mainstay of treatment, preventing further clot formation and promoting recanalization. *Salmonella *Typhimurium has been shown to induce thrombosis through activation of C-type lectin receptor-2 on platelets, highlighting a potential mechanism of infection-associated clot formation [11,12]. The Widal and Félix tests, commonly used for diagnosing typhoid fever, have moderate sensitivity and specificity, with false negatives often occurring in patients who have received prior antibiotic treatment [1].

Alternative diagnoses in our case, including viral and bacterial infections that could also cause CVT, were ruled out through a combination of imaging and laboratory tests. Negative CSF studies and clinical observation further supported the diagnosis of typhoid fever as the primary cause of the patient's condition.

Treatment typically involves empirical antibiotic therapy tailored to local resistance patterns, especially in cases of meningoencephalitis. Corticosteroids, when administered before antibiotics, may help reduce post-inflammatory scarring and improve long-term neurological outcomes by minimizing lasting deficits [13].

In our case, the patient showed a favorable response to a combination of antibiotics and anticoagulants. Thrombosis developed after gastrointestinal symptoms of *Salmonella* infection, indicating a potential immune-mediated mechanism. Although rare, systemic infections should always be considered as a potential cause of CVT in patients presenting with fever and headache, as early diagnosis and treatment are essential for preventing serious complications [14].

## Conclusions

This case highlights a possible association between* S. typhi* infection and CVT even in the absence of documented bacteremia. While a direct causal relationship cannot be confirmed, the systemic inflammatory response triggered by typhoid fever may contribute to a hypercoagulable state, potentially playing a role in the development of CVT. Given the rarity of this complication, it is crucial for clinicians to consider infectious causes such as *S. typhi* when evaluating patients with fever, headache, and altered consciousness, particularly when accompanied by gastrointestinal symptoms. Early recognition and appropriate treatment with antibiotics and anticoagulants are essential for a favorable outcome. This case underscores the importance of recognising CVT as a possible complication of typhoid fever. Timely and appropriate management may help minimise morbidity and prevent long-term neurological complications.
